# Early marriage, stressful life events and risk of suicide and suicide attempt: a case–control study in Iran

**DOI:** 10.1186/s12888-022-03700-0

**Published:** 2022-01-28

**Authors:** Ali Fakhari, Hamid Allahverdipour, Elham Davtalab Esmaeili, Vijay Kumar Chattu, Hamid Salehiniya, Hosein Azizi

**Affiliations:** 1 Research Center of Psychiatry and Behavioral Sciences, Tabriz University of Medical Sciences, Tabriz, Iran; 2 Department of Health Education and Promotion, School of Public Health, Tabriz University of Medical Sciences, Tabriz, Iran; 3 Road Traffic Injury Research Center, Tabriz University of Medical Sciences, Tabriz, Iran; 4 Department of Medicine, Temerty Faculty of Medicine, University of Toronto, Toronto, ON M5G 2C4 Canada; 5 Department of Public Health, Saveetha Medical College and Hospitals, Saveetha Institute of Medical and Technical Sciences, Saveetha University, Chennai, 600077 India; 6 Department of Community Medicine, Faculty of Medicine, Datta Meghe Institute of Medical Sciences, Wardha, 442107 India; 7 Social Determinants of Health Research Center, Birjand University of Medical Sciences, Birjand, Iran; 8 Department of Epidemiology and Biostatistics, School of Public Health, Tehran University of Medical Sciences, Tehran, Iran

**Keywords:** Early marriage, Stressful life events, Suicide, Suicide attempt, Case–control, Adolescents, Iran

## Abstract

**Background:**

Early Marriage (EM) and associated Stressful Life Events (SLEs) and consequences such as psychological and physical well-being issues can lead to suicide and suicide attempts (SA). The study aimed to investigate the risk of suicide and SA among early married people who experienced SLEs.

**Methods:**

A case–control study was conducted based on the registry for suicide in Malekan county in Iran during 2016–18. Cases included 154 SAs and 32 suicides. Simultaneously, 201 outpatients from the emergency department were chosen as controls. Holms and Rahe life event questionnaire was used to assess SLEs. Sub-group analysis (Mantel–Haenszel) by sex and age groups and multiple logistic regression were used to calculate adjusted Odds Ratios (ORs) with 95% Confidence Intervals (CIs) for the association between EM and suicide risk after adjusting for the potential confounders.

**Results:**

The proportion (female vs male) of EM among suicides, controls, and SAs was 31.25% (18.7 vs 12.5%), 15.92% (11.9 vs 4.0%), and 13.0% (11.7 vs 1.3%), respectively. In subgroup analyses by sex, EM was associated with an increased risk of suicide in both females and males 2.64 and 2.36 times, respectively. Likewise, subgroup analysis by age groups revealed that EM increased suicide risk in subjects aged 10–15 years, while no association was found for age groups of 26–40 and > 40. After adjusting for the potential confounders, EM (OR: 3.01; 95% CI: 1.15 -7.29), financial problems (OR = 4.50; 95% CI: 1.83 -9.07), and family problems (OR = 2.60; 95% CI: 1.19—9.59), were associated with an increased risk of suicide. However, no association was found between EM, various types of SLEs, and the risk of SA.

**Conclusions:**

We found EM and SLEs were correlated with suicide risk, while no evidence found that EM increased the risk of SA. Progress in reducing EM and addressing its serious consequences can occur by a stronger political commitment and by sharing the experiences and voices of the early married. Our study provided preliminary findings to guide future studies; however, methodological and longitudinal studies are needed to understand and address the effect of EM on suicidal behaviors.

## Background

Stressful life events (SLEs) are on the rapid rise in Iran and worldwide [1–3]. SLEs are ubiquitous, with 30% to 40% of the general population experiencing at least 1 major SLE [3]. SLEs and their various types, such as family conflicts and problems, are one of the main risk factors for mental disorders [3–6] and suicidal behaviors (SBs), which were highlighted in a meta-analysis study from Iran [7] and a population-based study in Denmark [8]. Evidence suggests that the history of SLEs could progress the risk of SBs and suicidal ideation [8, 9]. Suicide Attempters (SAs) and SBs have experienced many SLEs in their lifetime [10], and SLEs was related to suicidal ideation and overall suicidality in different age groups [11, 12]. Early marriage (EM), for the first time marriage, was a serious social issue in low-income countries that were associated with welfare, public health [13], and psychotic episodes [14] as one of the most important types of SLEs. Another study assessed serotonin 2A receptor role in the susceptibility to suicide and higher number of SLEs [15].

Early marriage, also known as child marriage, is the marriage or union between two persons in which one or both parties are younger than 18 years [16]. It is estimated that more than 21% of women married under the age of 18 around the world in 2020 [17]; in other words, almost 23 girl children got married each minute [18]. The highest rates of EM have been reported in Asian and African countries [19], particularly South Asia, including Bangladesh, India, and Nepal [18]. EM can affect girls’ psychological and physical well-being [20], so getting appropriate marriage timing is a fulcrum point for accessing outcomes related to fertility health, maternal and child nutrition, gender equity, livelihoods, education progress, and employment [21]. Accordingly, reduction in EM and delaying the marriage or appropriate age of marriage are included in an evidence-based package of suggested interventions addressing adolescent girls and boys [22, 23].

Although suicide and SBs mortality and morbidity are not common in most Islamic and Middle East countries, suicide has increased with a smooth slope in Iran in the last few years [24, 25]. Findings showed that the rate of suicide is common in the 15–35 age group in Iran [26]. The most common method is drug overdose or poisoning, and the highest suicide rate was reported in the summer season (35.2%) [27]. Among the Iranian population, the trend of 20-year suicidal deaths was increased with an average estimated rate of 9.9 per 100 000 persons [28]. Overall, 200 Years of Life Lost (YLL) per 100,000 persons due to SBs in Iran [29]. According to a health community assessment by the Malekan county health care system, suicide and SA (12 and 232 per 100,000) were recognized as the most important public health problems in 2013–14. Therefore, to reduce the burden of suicide and SA in the county, we developed and implemented a community-based suicide prevention program (Megaproject) in the PHC system [30].

EM has been identified as one of the most important social and public health problems in Malekan County that is associated with depressive symptoms and many other mental disorders. Another Iranian study showed that EM could progress to depressive symptoms [13]. On the other hand, depressive patients are more at-risk for suicide and SBs than the general people. Furthermore, previous findings highlight the impact of social determinants in women suicides in Iran and Muslim countries [31, 32] or a study provided family maladjustment related to women's self-immolation [33]. Family maladjustment and problems can have multiple roots. EM as one of the probable predictor factors of family maladjustment and problems (as a risk factor for suicidal behaviors) remains uncertain. As a result, identifying and gaining a better understanding of the impact of EM on suicide and SA would provide an effective perspective to health care system programs, especially in developing countries where the distribution of EM is common and conventional [34]. In Iran, the highest EM episodes were reported in Sistan and Baluchestan provinces (40%) [35]. The health programs can decrease a significant proportion of suicide and SA morbidity by moderating EM and SLEs issues. Likewise, the findings of this study can provide a framework for policymakers and health managers in developing an appropriate strategy for improving life skills and suicide prevention programs in adolescents and young people.

Although, various studies have been conducted on suicide in Iran [9, 30, 36–42]. However, the relationship between EM, SLEs, and the risk of suicide and SA is poorly understood, consistent with a small number of similar findings worldwide [10, 43]. Few studies explored EM implications, which are still unacceptably high in many countries, particularly in South Asia [8–10, 44, 45]. Hence, the present case–control study aimed to investigate the association between EM, SLEs, and risk of suicide and SA among married people in Iran.

## Methods

### Study design and sampling

We conducted a case–control study based on suicide and SAs records from the registry for suicide system, Malekan county bureau for mental health office during 2016–2018. This study was extracted from a suicide prevention project in Malekan County [30] and baseline data have been collected from it. Detailed methods of this community-based suicide prevention study have been published previously [25]. The study population was suicides or SAs in Malekan County. During this period, 32 completed suicide, and 642 SAs were registered in the county’s national registration system. We included all suicides (*N* = 32) during the study; however, 154 SAs were randomly selected and compared with 201 population-based controls to investigate the prevalence and associations between suicide, SA, and EM. Detailed methods and epidemiological aspects of suicide and SA among Malekan county people, East Azerbaijan province, and Iran have been published previously [24–26, 30].

The sample size was calculated using G-Power software 3.1.9.1 and previous studies in this county [9, 46] (α = 0.05%, β = 0.1, OR = 2, *P* = 0.25). After considering 10% to adjust for non-responders/ dropouts, the total sample size was estimated at 387 subjects.

### Case and control recruitment

The general population was the source population for both case and control subjects. Cases were suicides and SAs who registered in the national registry for suicide during the study period 2016–18. The national suicide registry system was developed in Iran in 2009 [29]. Suicides and SAs were compared with population-based controls with no history of SA or SBs during their lifetime.

For the cases from outpatients admitted to the county emergency ward to receive injury and trauma services in Farabi hospital (the only hospital in the County), we randomly chose controls simultaneously. Given that age distribution and demographic characteristics of controls were similar to the cases, and source population of both cases and controls were population-based registry system and general admissions, respectively. Non-natives, control participants with a history of SA or SBs, and subjects who refused to participate were excluded from the study.

### Measures

EM has occurred below 18 years at the first-time wedding or union between two persons. EM history was assessed before any suicide or SA events. We used native community-based health workers (*Behvarz* in Persian) who have face-to-face contact with large numbers of community members as part of their common routine practice [47] for collecting valid information about EM and SLEs status among all participants of the county.

The intensity of SLEs measured using a valid Persian version of the Holms and Rahe [48] life stress inventory instrument, which was used by our team in this study area previously [49–51]. This self-report events questionnaire has 43-items and measured the various common types of SLEs that happened to the respondents during the previous year. Respondents answered based on a relative score. Each event, called a Life Change Unit (LCU), had a different ‘weight’ for stress. More events mean a higher score. The higher the score and the larger the weight of each event, the more likely the patient would become ill. Here we mentioned some significant and reliable life stress events with a higher score, including the death of spouse or offspring (score: 100), Divorce (73), marital separation from a mate (65), (63), death of a close family member (63), major personal injury or illness (53), marriage (50), retirement from work (45), sexual difficulties (39), a major change in financial state (38), major change in a number of arguments with spouse (35), etc. The tool considered “marriage” a life stress event and pointed to a higher score = 50. Therefore, early marriage can be considered significant life stress.

The instrument has been validated by Roohafza et al., and the reliability of Cronbach's Alpha test was (α = 0.92) in Iran [52]. Also, Azizi et al. reported the reliability of the questionnaire was (α = 0.762) by Cronbach’s Alpha test [2].

A researcher-made questionnaire collected socio-demographic characteristics and the main predictors of suicide and SAs. This questionnaire was previously used by Azizi H et al. in this County for suicide prevention investigations [26]. A consensus discussion determined the validity among team members and authors, and the reliability was determined by the Cronbach's Alpha test (= 0.78) among 20 participants. The theoretical base for including the study variables was based on our community-based suicide prevention project in this county, for which data was collected previously [25, 26]. Socioeconomic indicators included educational level, family (household) income, and occupation/employment status. Gross monthly household income from all sources was categorically measured based on Iranian currency (Rials): less than 10, 10–20, 20–30, > 30 million Rials. Educational level was measured based on educational years and grouped as primary school, secondary school, high school, and academic. Occupation/employment status was measured by an open question in the interviews and categorized on students of the school and college (as a frequent group of SA in this County), housewife, farming or farming-related (a typical job in this area), self-employed, and unemployed.

Participants were invited to the health center, and then questionnaires were completed in a face-to-face position by trained interviewers. The interviews were performed in a single sitting for all participants.

### Statistical analysis

The SPSS software (version 19.0, Chicago, IL, USA) was used for data analysis. Data normality assessed by Kolmogorov–Smirnov test. Bivariate analysis was utilized as the first step to compare control and case groups using appropriate tests (t-test for quantitative and chi-square (χ2) test for dichotomous variables). The control group was considered baseline/reference category, and then SAs and suicide cases (case group) were compared with controls. The distribution of EM was compared among study groups (suicides, SAs, and controls) by sex type and age groups.

### Adjusting the effect of sex and age groups for estimating the association between EM and suicide

Chi-square (χ2) test by using Mantel–Haenszel analysis was used for adjusting the impact of gender (female and male) and age groups as the potential confounders to estimate actual and adjusted Odds Ratios (ORs) for the association between EM and risk of suicide and SA.

### Multiple regression analyses

All variables with a p-value less than 0.2 were analyzed using the backward stepwise method with multiple logistic regression. The Hosmer and Lemeshow statistic was used for checking data matching and goodness of fit with the model. Age (groups), sex, educational level, and family income were adjusted for estimating adjusted ORs with 95% Confidence Interval (CIs) for the relationship between EM, SLEs, and suicide risk. In all tests, *p*-value < 0.05 was considered significant.

## Results

Of the total 387 subjects, a majority (114, 57%) were females. Among completed suicides, almost 72% were males, while in SAs cases, most participants were female (64%). Age and sex had a significant relationship with the risk of suicide (*p*-value < 0.05). Regarding occupation and family income, a significant association was observed between groups. Furthermore, among behavioral risk factors, we found an association between suicide and alcohol abuse, smoking, and a history of depression (Table 1).

**Table1:** Comparison of socio-demographic characteristics, depression, and selected behavioral risk factors between case and population-based control groups

Variables		Control (*n* = 201)	Case (*n* = 186)		P-value
		Without SB	Suicide Attempters (*n* = 154)	Suicide (*n* = 32)	
**Gender**	Female	114 (56.7)	99(64.28)	9(28.12)	0.002
	Male	87 (43.3)	55(35.72)	23(71.9)	
**Age**	10–25	93 (46.25)	95(61.69)	9(28.125)	0.001
	26–40	61 (30.35)	45(29.22)	18(56.25)	
	≥ 40	47 (23.38)	14(9.1)	5(15.63)	
**Occupation**	Student	46 (22.88)	34(22.08)	7(21.87)	0.034
	Farming related	17 (8.45)	4(2.6)	2(6.25)	
	Household	94 (46.76)	102(66.23)	5(15.63)	
	Others	44 (21.9)	14(9.09)	18(56.25)	
**Marital status**	Single	42 (20.9)	26(16.88)	10(31.25)	0.028
	Married	144 (71.65)	115(74.67)	21(65.63)	
	Widow and Divorced	15 (7.46)	13(8.45)	1(3.12)	
**Educational level**	Primary school	65 (32.34)	52(33.76)	10(31.25)	0.247
	Secondary school	109 (54.23)	80(51.95)	19(59.38)	
	High school and Academic	27 (13.43)	22(14.28)	3(9.37)	
**Family size**	2 ≥	35 (17.42)	29(18.83)	4(12.5)	0.317
	3–4	106 (52.73)	83(53.9)	16(50.00)	
	≥ 4	60 (29.85)	42(27.27)	12(37.5)	
**Income (million rials)**	≤ 10	67 (33.33)	72(46.75)	8(25.00)	0.029
	10 – 20	72 (35.82)	57(37.01)	13(40.62)	
	20 – 30	77 (38.3)	17(11.04)	4(12.5)	
	≥ 30	62 (30.85)	8(5.19)	7(21.88)	
**Resident**	Urban	56 (27.86)	31(20.13)	2(6.25)	0.334
	Rural	145 (72.14)	123(79.87)	30(93.75)	
**Life alone**		10 (4.98)	8(5.2)	1 (3.12)	0.530
**History of Depression**		49 (24.37)	45 (29.22)	3 (9.37)	0.030
**Substance abuse**		8 (4)	4 (2.6)	2 (6.250	0.347
**Alcohol abuse**		20 (9.95)	9 (4.48)	11 (34.37)	0.001
**Smoker **^**a**^		30 (14.92)	17 (8.45)	3 (9.37)	0.001

^a^at least 2 cigarettes per day

Table 2 shows the prevalence of EM and subgroup analyses of crude and adjusted (Mantel–Haenszel) ORs with 95% CIs for the association between EM and suicide risk by sex and age groups. The prevalence of EM in women was higher than males in all study groups. The overall prevalence (gender-based: female vs male) of EM was 31.25% (18.7 vs 12.5%), 15.92% (11.9 vs 4.0%), 13.0% (11.7 vs 1.3%) among suicides, controls, and SAs, respectively. In subgroup analyses by sex, EM marriage was associated with an evaluated risk of suicide in both females and males 2.64 and 2.36 times, respectively. After adjusting for the gender as the potential confounder by the Mantel–Haenszel model, EM increased risk of suicide adjusted OR = 2.41 (95% CI: 1.40–6.67). However, there was no significant association between EM and risk of SA in subgroup analyses by sex, as well as in crude and adjusted ORs (Table 2).

**Table 2:** Early Marriage prevalence (EM) and subgroup analyses of crude and adjusted (Mantel–Haenszel) Odds Ratios (ORs) with 95% CIs for the association between EM and suicide risk by sex and age groups

Variables				Controls (*n* = 201)	Suicide attempters (*n* = 154)	Suicides (*n* = 32)	Comparison of suicides and controls		Comparison of SAs and controls OR (95% CI)	
Age groups	Mean ± SD			26.21 ± 9.76	26.08 ± 9.87	2.8 ± 10.21	Crude OR (95% CI)	Mantel–Haenszel OR (95% CI)	Crude OR (95% CI)	Mantel–Haenszel OR (95% CI
	*10–25*	EM	*No*	78 (38.8)	81(52.6)	4 (12.5)	3.44 (1.63–7.26)	^a^2.21 (0.95–5.16) 0.061	0.90 (0.40–1.98)	0.70 (0.37–1.30) 0.261
			*Yes*	15 (7.5)	14 (9.09)	5 (15.6)				
		*P-value*					0.013		0.475	
	*26–40*	EM	*No*	48 (23.9)	39 (25.3)	14(43.7)	1.04 (0.39–2.80)		0.57 (0.20–1.63)	
			*Yes*	13 (6.5)	6 (3.9)	4(12.5)				
		*P-value*					0.583		0.212	
	> *40*	EM	*No*	43 (21.4)	14 (9.09)	4 (12.5)	2.35 (0.32–7.15)		1.12 (0.85–1.52)	
			*Yes*	4 (2.0)	0 (0.0)	1 (3.12)				
		*P-value*					0.410		0.342	
Sex	*Female*	EM	*No*	90 (44.7)	81 (52.6)	4 (12.5)	2.64 (1.33–5.23)	^b^2.41 (1.40–6.67) 0.008	0.83 (0.42–1.64)	0.72 (0.39–1.33) 0.297
			*Yes*	24 (11.9)	18 (11.7)	6 (18.7)				
		*P-value*					0.033		0.363	
	*Male*	EM	*No*	79 (39.3)	53 (34.4)	18 (56.2)	2.36 (0.85–6.56)		0.37 (0.08–1.82)	
			*Yes*	8 (4.0)	2 (1.3)	4 (12.5)				
		*P-value*					0.102		0.179	
	*Total*	EM	*No*	169 (84.08)	134 (87.0)	22 (68.75)	2.40 (1.07–5.54)		0.78 (0.43–1.44)	
			*Yes*	32 (15.92)	20 (13.0)	10 (31.25)				
		*P-value*					0.038		0.268	

^a^The association between EM and suicide after adjusted for the “age groups”

^b^The association between EM and suicide after adjusted for the “sex”

Concerning the prevalence rates of EM among suicides by age groups, the age group of 10–25 (15.6%) in suicide cases had the highest prevalence of EM than age groups of 26–40 (12.5%) and > 40 (3.12%). Subgroup analyses for the association between EM and suicide risk by age groups indicated that EM increased suicide risk in 10–15 years. We did not find significant associations between EM and suicide risk in age 26–40 and > 40 years. After adjusting for age groups as the potential confounding, EM marriage insignificantly associated with suicide adjusted OR = 2.21 (95% CI: 0.95–5.16). Furthermore, we found no evidence for the association between EM and SA in subgroup and adjusted analyses (Table 2).

Table 3 indicates the relationship between various types of SLEs and the risk of suicide and SA among case and population-based control groups. Family problems, a major change in number of arguments with spouse, financial problems, and exposure to the new conditions were significantly associated with suicide (*p*-value < 0.05).

**Table 3:** Associations between SLEs and risk of suicidal behaviors

Variables	Controls (*n* = 201) (*n* = 201)	Case (*n* = 186)		P-value
		Suicide Attempters (*n* = 154)	Suicide (*n* = 32)	
**Family problems**	59 (29.35)	38(24.67)	19(59.38)	0.001
**Major change in number of disagreements with spouse**	86 (42.78)	59(38.31)	19(59.38)	0.008
**Loss of loved ones**	41 (20.4)	31(2013)	4(12.50)	0.385
**Financial problems**	25 (12.43)	10(6.50)	15(46.88)	0.001
**Life failures**	9 (4.48)	7(4.55)	2(6.25)	0.530
**Emotional problems**	20 (9.95)	9(5.84)	8(25.00)	0.003
**Expose to the new conditions**	5 (2.48)	3(1.95)	3(9.37)	0.064
**unemployment of > 6 months**	30 (14.93)	22(14.28)	8(25.00)	0.187

Regarding the correlation between suicide, SA, and the number of SLEs, Table 4 demonstrates the trend and association between the number (frequency) of SLEs with suicide and SA. There was a significant trend between increasing the number of SLEs and the risk of suicide (*p* = 0.001). Participants without SLEs were considered the reference group, and then SAs and suicides were compared with those. We found individuals who completed suicide and SAs were experienced more SLEs than the control group, and it had increased odds of suicide and SAs risk, respectively (AOR: 8.86, 95% CI: 3.29–19.06), AOR: 6.52; 95% CI: 2.28–13.65).

**Table 4:** The frequency of SLEs among study groups

**Number of SLEs**^**a**^	**Controls**	**Suicide attempters**	**Suicide**	**Comparison of suicide and control** Adjusted OR (95%CI)	**Comparison of suicide attempted and control** Adjusted OR^a^ (95%CI)
0	133 (66.2)	11 (7.15)	1 (3.1)	1.00^b^	1.00^b^
1 – 2	61 (30.35)	94 (61.04)	14 (43.7)	4.47 (2.83–13.62)	2.94 (1.27–10.77)
≥ 3	7 (3.5)	49 (31.81)	17 (53.2)	8.86 (3.29–19.06)	6.52 (2.28–13.65)

^a^Cochran-Armitage trend test, *P* > 0.001

^b^Reference group

**Fig. 1:**
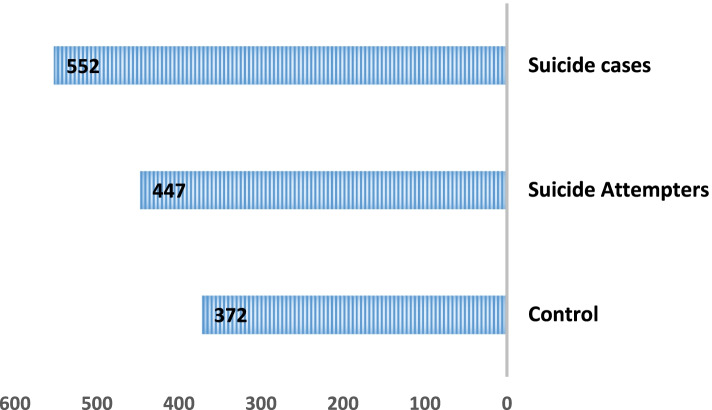
Comparison* of mean scores of SLEs ** among three groups of suicides, suicide attempters, and population-based controls. *ANOVA, *P* = 0.001. **SLEs score (Mean ± SD): Controls: 392 ± 33.26, Suicide attempters: 447 ± 27.06, suicide: 552 ± 23.75

Figure 1 shows the mean and standard deviation of SLEs scores (in the last year) among three groups of study participants: suicides, SAs, and controls. Suicide cases had the highest SLEs score (552 ± 23.75). A significant difference also was found between SLEs score and suicide risk (ANOVA, *p* = 0.001).

Table 5 demonstrates multiple logistic regression analyses for estimating adjusted odds ratios and 95% CIs for the association between EM, various types of SLEs, and risk of suicide and SA.

**Table 5:** Adjusted^a^ ORs with 95% CIs for the association between EM, SLEs and risk of suicide and SA by multiple logistic regression analyses

Variables	Comparison of suicides and controls	P-value	Comparison of attempters and controls	P-value
	OR (95% CI)		OR (95% CI)	
**Early marriage**	3.01 (1.15 -7.29)	0.025	1.40 (0.75—2.60)	0.286
**Family problems**	2.60 (1.19—9.59)	0.038	1.12 (0.68—1.90)	0.627
**Financial problems**	4.50 (1.83 -9.07)	0.001	2.04 (0.95—4.39)	0.067
**Expose to the new conditions**	1.32 (0.84 -5.86)	0.180	0.83 (0.53—1.39)	0.419

^a^Adjusted for age, sex, educational levels, and family income per month

After adjusting for the potential confounders (sex, age (groups), educational level, and family income), multiple regression analysis showed a significant association for suicide risk by EM (OR: 3.01; 95% CI: 1.15 -7.29, *p* = 0.025), financial problems (OR = 4.50; 95% CI: 1.83 -9.07, *p* = 0.001), and family conflicts (OR = 2.60; 95% CI: 1.19—9.59, *p* = 0.038), while, we found no significant associations between EM, various types of SLEs and SA risk.

## Discussion

After adjusting for the potential confounders, we found that EM and SLEs were associated with suicide risk. However, no evidence found that EM increased the risk of SA. Our results indicated that suicides had the most prevalence rate of EM and SLEs than population-based controls. Further, our findings demonstrated that EM episodes and various SLEs are common among suicide cases. Concerning that EM is common among females, the gender distribution may confound the actual association between EM and suicide risk. Sub-group analyses adjusted the gender variable, and there were significant associations between EM and suicide risk in both female and male genders. Likewise, subgroup analysis by age groups revealed that EM increased suicide risk in subjects aged 10–15 years, while this association was not significant for age groups of 26–40 years and > 40 years. These findings are significant by two aspects of demonstrating the association between EM and suicide. First, it shows the sex and age distribution agreement between EM and suicide deaths, given that EM is defined under 18 years. Second, the short time interval between EM and suicide, the more likely they were to be related since suicide is often an acute event. Although, EM can lead to long-term marital conflicts and its effect on suicide. However, the present study confirms the first hypothesis.

Moreover, to ascertain valid evidence for the association between EM and suicide, in the final analyses by multiple logistic regression, we adjusted variables including educational level, family income, sex, and age groups for the actual association between EM and suicide and SA risk. These four variables had a causal relationship between EM and suicide, notably educational level and female sex. Educational level directly affects EM prevalence, and academic and high educational levels reduce EM statistics. However, high education does not guarantee suicide risk reduction. Therefore, investigations about EM and suicide risk deserves attention and further investigation.

Unlike suicide, we did not find evidence for increasing SA risk by EM upon sub-group and multiple logistic regression analyses. The prevalence rate of EM among SAs and population-based controls were almost equal. This difference seems to be related to the nature and casuals of suicide and SA.

These findings highlight the need to consider a wide range of contextual and sociocultural factors on suicide prevention strategies suggested in the community-based studies, at least in Iran [53].

EM is a common social issue and one of the negative life events that deserve attention; especially, it can influence psychological well-being and the quality of life worldwide [1]. Addressing the EM issue will provide considerable evidence regarding the characteristics of suicide and their quality of life. It will help enhance the strategies and policies that may be used to help child/early married girls and boys have better and more meaningful lives. Previous research has revealed that people with an EM background are more likely to have marital conflicts and depressive disorders, especially if they were forced to marry or one of the partners was unhappy in their marriage [54–56]. EM was identified as one of the potential risk factors of suicide in this study. Several studies in Iran, including ours, found that family conflicts significantly predict SAs and suicide [54–56].

Evidence reported that many family problems and arguments correlate among EM people [57]. Findings suggested that EM individuals were unhappy with the present marriage. Although the gender differences were reported in SA [58], husband dissatisfaction leads to marital and family conflicts or psychological as a major risk for suicidal behaviors [59]. Therefore, family disagreements and depression as the most important determinants of suicide and SA are associated with EM. Moreover, longitudinal and methodological investigations are needed to ascertain valid information and causal inference. However, another reason may be this county's different cultural and social status, as suicide determinants are different in various settings and are strongly influenced by local culture and religion.

In this study, almost 70% of the county’s population lives in rural areas with poorly educated. It is possible, living in rural areas and poor education and being distant from the academic settings are the main reasons for EM in this county [60] however, additional research is required. In addition, EM was correlated with suicide; it is also associated with adolescent mental health, poor quality of life, marital skills, well-being, academic progress or education cessation, and their offspring’s health. It also can prevent ideal health utility. However, EM can elevate the possibility of sexually transmitted diseases, cervical cancer, and death at delivery [13, 35, 61].

Although suicide and SA aren’t common among Islamic countries, the prevalence of suicide has increased over the past decades in Iran [24]. Despite the side effects of EM on suicide and mental health, limited evidence exists on the relationship between EM and suicide and SA risk [62–64]. This study is the first investigation in Iran that assessed the association between EM and SLEs with suicide risk among three groups of participants (suicides, SAs, and population-based controls) [9]. In agreement with our findings, the global studies reported SLE and the risk of suicide or SA, including studies in China [65, 66] and a population-based study in Denmark [8]. Most Western societies have also examined SLEs among SA or suicidal cases [8, 67, 68].

Nevertheless, our study assessed the impact of both suicide and SA risk by comparing a population-based control group [66, 69]. Besides, a meta-analysis study has reported that SLEs are associated with suicidal risk [70]. Studies that have not found an association between suicide and SLEs are limited, including a study from India that did not find a relationship between suicide and SLEs. Still, they reported that SLEs are associated with more help-seeking behavior [71].

Another type of SLEs, financial problems were associated with an increased risk of suicide. In this study, suicide and SA cases had more financial problems than controls. This relationship was also observed with the family economic levels. In our study, individuals with less than 10 million Rials income per/month had the highest frequency of SA. An Iranian study by Ahmadi et al. identified financial problems as one of SB's main determinants and risk factors [72]. Other findings show a significant and positive relationship between financial problems, unemployment, income inequality, and suicide [73–75]. As a result, efforts to reduce unemployment and income inequity will have a significant role in suicide prevention.

Therefore, the impact of SLEs is highly significant on developing suicide and SA especially social issues such as EM. It is believed that prevention and control of suicide requires a multifaceted approach incorporating active collaboration of the health system, government, legal sectors, social welfare, and urban/rural development. Therefore, it is suggested that community-based suicide prevention strategies and intervention programs should be started to prevent suicide and SA and their predictors, especially EM and SLEs, through developing a comprehensive policy [76]. Evidence suggested that Primary Health Care (PHC) could be an effective and feasible position for developing suicide prevention programs [30].

Developing proper care and monitoring programs can help strengthen and enhance several elements of adolescent and young adult life skills and avoid EM and suicide. Given that, suicide prevention benefits persons and families and profits the health care system and the welfare of communities and society at huge [13, 77].

### Limitations

Our study had some limitations. EM marriage tradition was common in this county, and it was one of the main reasons for conducting the present study. However, establishing casual inference and measures of connections between suicide or SA and EM is challenging and should be done with caution because suicide and SA are affected by various personal and environmental factors. It is possible that EM was located an intermediate variable in the causal path between risk factors and suicide. To resolve this problem, first, we assessed EM history before any SA or suicide via face-to-face interviews and using community health workers (*Behvarz* in Persian). In the second stage, the association between EM and suicide was stratified by sex and age groups.

Given that suicide is an acute event, the time interval between EM and death by suicide should be narrow. Our stratified analysis indicated that the age distribution of EM was compatible with the age of suicide cases, and the majority of EM and suicide have occurred among those aged 10–26 years. Furthermore, gender-based results found EM was associated with suicide in both females and males.

However, we suggest longitudinal and methodological studies for long-term follow-up measures to confirm the implications of EM on suicide and SA in the various settings and communities. Although, in a recent survey [1] a significant relationship was found between EM and depressive disorders among girls and boys how suffered from child marriage.

Furthermore, differentiating forced marriages from early marriages and EM episodes in one or both married people can assist future studies in giving accurate evidence about EM and suicide or SA. We investigated and compared the impact of SLEs between suicide and SA. People who completed suicide were unavailable. We tried to reduce this problem by using community health workers (*Behvarz* in Persian) who have face-to-face contact with a large number of community members as part of their usual routine performance and interviews with family members, including parents, spouses, or siblings.

## Conclusions

We found EM and SLEs were correlated with suicide risk, while no evidence found that EM increased the risk of SA. Our study provided preliminary findings to guide future studies. Methodological and longitudinal studies are needed to understand and address EM's role in suicide and SA, well-being, and quality of life. It is recommended that the effect of EM episodes in one or both married persons and the impact of forced marriages should be investigated separately to provide valid evidence about EM and suicide or SA in future studies.

## Data Availability

The datasets generated and/or analyzed during the current study are available from the corresponding author on reasonable request.
